# Tyrosine kinase inhibitors enhanced the efficacy of conventional chemotherapeutic agent in multidrug resistant cancer cells

**DOI:** 10.1186/s12943-018-0775-3

**Published:** 2018-02-19

**Authors:** Shaocong Wu, Liwu Fu

**Affiliations:** State Key Laboratory of Oncology in South China, Guangdong Esophageal Cancer Institute; Cancer Center, Sun Yat-sen University Cancer Center, Guangzhou, 510060 China

**Keywords:** Tyrosine kinase inhibitor, ABC transporter, Multiple drug resistance, Chemotherapy, Chemosensitizer

## Abstract

Multidrug resistance (MDR) triggered by ATP binding cassette (ABC) transporter such as ABCB1, ABCC1, ABCG2 limited successful cancer chemotherapy. Unfortunately, no commercial available MDR modulator approved by FDA was used in clinic. Tyrosine kinase inhibitors (TKIs) have been administrated to fight against cancer for decades. Almost TKI was used alone in clinic. However, drug combinations acting synergistically to kill cancer cells have become increasingly important in cancer chemotherapy as an approach for the recurrent resistant disease. Here, we summarize the effect of TKIs on enhancing the efficacy of conventional chemotherapeutic drug in ABC transporter-mediated MDR cancer cells, which encourage to further discuss and study in clinic.

## Background

Cancer, a group of diseases involving abnormal cell growth with the potential to invade or spread to other parts of the body, has developed as second leading cause of disease-related death [1, 2]. Lots of options served as anti-cancer therapy exist. Among them, chemotherapy applies the most. Indeed, the prescription of chemotherapeutics is such an brilliant success that should be regarded as a milestone in anti-cancer career. However, despite chemotherapeutic outstanding performance treated in cancer, there are accumulating and clear evidences of acquired drug resistance, especially MDR to them [3, 4], a phenomenon that cancer cells once exposed to one anti-cancer drug show resistance to various other drugs that are structurally and functionally different from the initial anti-cancer drug, impairing drug efficacy and accounting for 90% deaths in cancer. A great deal of researches reveals the potential mechanism conferring MDR in chemotherapy, including kinase domain gene mutation [5], target gene amplification, the modification of signal pathway and the activation of parallel ones [6, 7]. Among these, ABC transporter, driven by ATP hydrolysis, plays an essential role in the genesis of MDR, especially ABCB1 [8], ABCC1 [9] and ABCG2 [10]. They’re expressed constitutively in both cancer and normal cells, participating in the process of absorption, distribution, metabolism, excretion and toxicity (ADME-Tox) [11]. ABC transporter has documented as an efflux pump for multiple anti-tumor drugs, which decreases the intracellular drug concentrations and leads to MDR phenotype, implying the modulators of ABC transporter might potentially be applied in MDR cancer cells and act as chemosensitizers, such as verapamil, PSC-833 and GF120918 [12]. TKIs, also called tyrphostine, a series of pharmaceutical drugs that suppress ATP-binding site of tyrosine kinase, function as a target-specific remedy in anti-cancer regimen [13]. As far, at least 20 TKIs aiming to various tyrosine kinase, e.g. EGFR, VEGFR, PDGFR [14], have been generated proven to be effective anti-tumor agents clinically which received the Food and Drug Administration (FDA) approval [15]. Coincidently, just like tyrosine kinase, ABC transporters happened to have the ATP-binding site. It is conceived that TKIs might be inhibitors of ABC transporters as well as tyrosine kinase. Consequently, an increasing number of testimonies lied on this assumption show numerous TKIs could function as inhibitors of ABC transporter, hence hamper the efflux of anti-cancer drug and promote the intracellular accumulation of them, indicating that TKIs seems to be chemosensitizers in MDR and enhance the efficacy of chemotherapeutic agents by combinational therapy [16–19].

In this present review, we struggle to demonstrate the application of small molecule TKIs and related remedy in the clinical, the relationship between ABC transporter and MDR, as well as the ongoing or accomplished pre-clinical and clinical researches regarding to TKIs’ new-found function as MDR chemosensitizers when combined with conventional chemotherapeutic agent and the underlying mechanism on it. We sincerely hope that the information involved here could serve as references to overcome MDR and diminish unnecessary side effect, ultimately optimize the treatment in anticancer therapy.

### ABC transporters and ABC transporter modulators

#### ABC transporters

ABC transporters, a family of membrane protein, consist of 48 members identified in humans which are classified into 7 subfamilies labeled A-G [20, 21]. They express constitutively in both cancer and normal cells, functioning as importers or exporters [22] and subsequently influencing the process of absorption, distribution, metabolism, excretion and toxicity (ADME-Tox) [23, 24]. Structurally, most ABC transporters consist of 2 transmembrane domains (TMDs, which span the membrane and form a channel) and 2 nucleotide-binding domains (NBDs, where bind and hydrolysis ATP via ATPase) [25]. Due to the limited scope of this review, we just take ABCB1 for example in order to explain how ABC transporter work as a pump. In the absence of ATP binding to NBDs, the 2 TMDs form a barrel-like shape with a central pore that is open to the extracellular surface and spans much of the membrane depth allowing no substrate to get through it. Once ATP binds to NBDs, driven by the energy of ATP hydrolysis, TMDs initiate the conformational change and consequently form a channel in manner that could allow access of substrates directly transport from one side of cell membrane to another [18], leading to the alteration of ADME-Tox. Despite a diverse array of substances could be transported by ABC transporters, including lipids [26], amino acids [27], sugars [28], peptides [29] and numerous drugs [30], there are documented evidences that ABC transporters exhibit a characteristic property of relative selectivity and specificity, which means different kinds of ABC transporters might carry only their own substrates, indicating their completely distinct function [21]. Among of all ABC transporters, the ABCB1, ABCC1 and ABCG2 have been intensively studied because of their crucial roles in the genesis of MDR [31–36].

#### ABC transporters and MDR

**Fig. 1:**
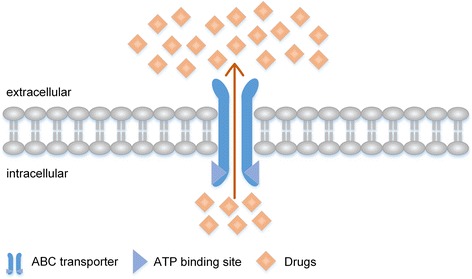
ABC transporters decrease intracellular drug concentration conferring MDR. Cancer cells promote overexpression or activation of ABC transporter, enhancing the efflux of chemotherapeutic drugs, which leads to a lower intracellular drug concentration and results in MDR phenotype

Simultaneous resistance of cancer cells to multiple anti-neoplastic agents that are structurally and functionally unfamiliar is known as MDR. Cancer cells with the MDR phenotype may have either inherent resistance to anti-cancer drugs or resistance acquired after cycles of chemotherapy. Intrinsic or acquired MDR is one of the main reasons for chemotherapy failure, leading to the recurrence of malignant tumors and ultimately, patient relapse or death. As we know, ABC transporters have been documented as a pivotal role in MDR phenotype [37, 38]. Functionally, ABC transporter can pump chemotherapeutic drug out of cancer cells, decrease intracellular accumulation of anticancer drug and result in cancer cell resistance (Fig. 1) [39].

#### ABC transporter modulators

Given that the close relationship between overexpression of ABC transporters and MDR phenotype, lots of drugs which can inhibit the activity of ABC transporter, namely ABC transporter modulators, have been found in order to reverse MDR. Until today, ABC transporter modulators’ development has been through several distinct generation and could be classified into four categories according to their strategy employed in discoveries (Table 1): (i) the first generation modulators, such as verapamil, cyclosporin A and tamoxifen, were found effective in vitro while exhibited an upset outcome in vivo because of their low affinity to ABC transporter and unacceptable toxicity [40]. (ii) the second generation modulators, including PSC833, S97882, had been proven effective both in vitro and in vivo along with relative mild toxicity when compared with the first [41, 42]. But they interacted with conventional chemotherapeutic drug in pharmacokinetics, which resulted in unpredictable side effect in clinic. (iii) the third generation modulators, e.g. GF120918 and XR9576, unlike the first and second generation, were shown less influence on pharmacokinetics which means they might be applied in MDR cancer patients without severe systemic toxicity [43, 44]. (iv) the fourth generation modulators, such as neochamaejasmin B (NCB) and curcumin, sharing a property of less toxicity and better oral bioavailability compared the former generations. It was reported that they potently reversed MDR by down-regulating the expression of ABC transporters [45, 46]. A number of clinical trials of MDR modulators have been conducted in various different cancers types. Unfortunately, almost no substantial survival benefits have been established, which has largely limited their widespread clinical application. To find novel and potent MDR modulator is still key issue to overcome MDR.

**Table 1:** The characteristics of 4 generations of ABC transporter modulators

**Generation**	**Representative**	**In vitro**	**In vivo**	**PK**	**Down-regulate ABC transporter**
1st	verapamil, cyclosporin A, tamoxifen	+	–	+	–
2nd	PSC833, S97882, VX-710	+	+	+	–
3rd	GF120918, XR9576, R101933, LY335979	+	+	–	–
4th	neochamaejasmin B, curcumin	+	+	–	+

“+” stands for positive effective; “-” stands for ineffective; “PK” stands for pharmacokinetics

### Tyrosine kinase and tyrosine kinase inhibitors

#### Tyrosine kinase

Protein tyrosine kinase (PTK) is a series of enzyme that can transfer a phosphate group from ATP to a protein in a cell [47], which acts as a important role in the genesis of cancer through abnormal transduction [48, 49]. PTKs could be classified into 2 families: receptor tyrosine kinase (RTKs) and non-receptor tyrosine kinase (NRTKs) [50]. The structure of RTK can be divided into three parts [51]: (i) an extracellular ligand-binding domain (ii) a transmembrane-spanning region (iii) an intracellular catalytic domain. RTK is presented as a monomer without activation by binding to exclusive ligand [52]. Once binding to a specific ligand, it will induce homo/hetero-dimerization of the receptor, leading to a conformational change which results in cross-phosphorylation of tyrosine residues. Consequently, the activated phosphorylated residues will assemble as a signaling complex which initiates a cascade of intracellular signaling pathways and interferes cellular proliferation and survival [53]. Abnormal uninterrupted activation of it might disequilibrate signal transduction and transform a cell from a normal state into a cancerous one. Compared to the RTKs, NRTKs are cytoplasmic enzymes, indicating an absence of the extracellular domain and transmembrane-spanning region. Actually, NRTKs can be regarded as downstream factors triggered by RTKs and share a similar mechanism of oncogenesis. Since PTK share a close relationship with tumorigenesis, whether the inhibitor of it could exhibits anticancer effect is engrossing [54, 55].

#### Tyrosine kinase inhibitors

**Fig. 2:**
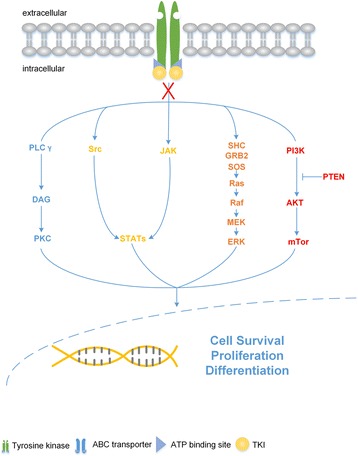
TKIs inhibit TKs-mediated signaling pathway. TKIs inhibit tyrosine kinase, consequently interrupting the subsequent downstream signaling pathways, influencing cell proliferation and differentiation and leading to the arrest of cell growth

An accumulated researches and recognition involving to the critical role of tyrosine kinase in tumorigenesis have raised scientists’ awareness to focus on inhibitor of tyrosine kinase [56–58], of which constitute a main component of the pipelines of oncology drug development [59]. Until today, there are at least 20 TKIs receiving FDA approval and functioning as anticancer drugs [60], while numerous are in the process of pre-clinical or clinical trials. Mostly TKIs compete with ATP to bind to the intracellular catalytic domain of tyrosine kinase and consequently inhibit the process of cross-phosphoralation which is essential to the activation of TKs and the formation of signaling complex [59, 61], interfering the subsequent downstream signaling pathways, then impairing cell proliferation and survival, which leads to the arrest of cell growth (Fig. 2) [62–64]. In 2001, imatinib, the first TKI approved by FDA prescribed to CML, a kind of blood cancer performing a new fusion gene BCR-Abl which encodes a cytoplasm-targeted tyrosine kinase, had received brilliant success [65]. Unfortunately, though TKIs exhibit a promising potence in anticancer therapy, an increasing evidences show that cancer cells treated with TKIs tend to acquire drug resistance which will impair the efficacy of these target-specific agents [6, 66]. In order to circumvent drug resistance when received TKIs therapy, we take EGFR TKIs for example, four generations of them were developed, depending on their clinical strategies (Table 2) [67, 68]. On the other hand, most TKI was used alone in clinic while drug combinations acting synergistically to kill recurrent resistant cancer cells have become more and more important in cancer chemotherapy.

**Table 2:** The comparison of 4 generation of EGFR TKIs

GN	RP	WT	Gene mutation			BI
			Exon 19/21	T790 M	C797S	
1st	Icotinib, Gefitinib, Erlotinib	+	+	–	–	–
2nd	Afatinib, Dacomitinib	++	+	+	–	+
3rd	Osimertinib, Rociletinib, Olmutinib, Nazartinib, Avitinib, ASP8273, PF-06747775, HS-10296	–	++	++	–	+
4th	EAI045	–	++	++	+	+

“+” stands for effective; “-” stands for ineffective; “GN” stands for generation; “RP” stands for representative; “BI” stands for binding irreversibly

### Interaction of TKIs with ABC transporters

To date, in order to figure out the interaction between TKIs and ABC transporters which could predict the ADME-Tox properties of drugs and forecast anticancer efficiency of TKI in ABC transporter-mediated MDR background, ABC transporters and TKIs are intensively investigated. Unfortunately, because of a lack of direct correlation between them, all we know is a phenomenon that most TKIs are endowed of substrate-like property at low concentration while at high concentration they likely act as inhibitors of ABC transporters [69]. Due to the scope limitation, then we will mainly concentrate on their inhibitor-like property.

#### ABC transporters extrude TKIs, potentially conferring TKI resistance

As mentioned previously, though TKIs have higher selectivity and milder toxicity when compared to conventional chemotherapeutics, the occurrence of TKI resistance has been extensively reported. Mechanisms conferring TKI resistance are varied, overexpression of ABC transporters represents one of them [11]. As a pump, ABC transporter can extrude various substances, including TKIs, which leads to drug resistance. Imatinib, prescribed in CML patients, was firstly reported of ABC transporter-mediated TKI resistance by Mahon and colleagues in 2000 [70]. They initiated a experiment aimed at establishing the functional relevance of STI571 with ABCB1 and finally concluded that CML cell line overexpressing ABCB1 would impair the uptake of STI571 and confer resistance to Imatinib. It’s necessary to note that numerous TKIs approved by FDA were reported of ABC transporter-mediated resistance until now, such as Nilotinib, Sunitinib, Gefitinib, Erlotinib and Lapatinib [71, 72]. Limited by the scope, we won’t state in detail here. In general, resistance to TKIs attenuate the anticancer efficiency and impair patients’ outcome. Elucidation of interaction between TKI resistance and ABC transporter could predict cancer patient’s prognosis when treated with TKI.

#### TKIs inhibit ABC transporters, potentially functioning as chemosensitizers

**Fig. 3:**
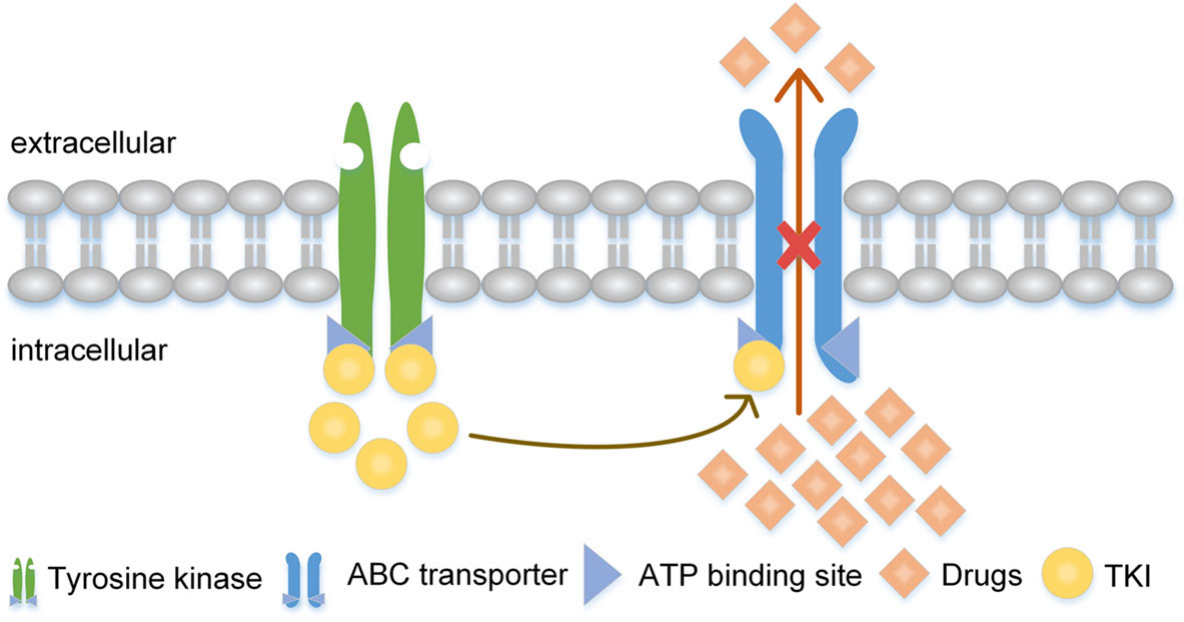
TKIs inhibit ABC transporters. Both TKIs and ABC transporters compose of ATP-binding site. TKIs connect to ATP-binding site of ABC transporters and inhibit its function of discharging anticancer drugs out of MDR cells

As the MDR phenotype prevails, there is an urgent need to develop new strategies to circumvent it. One’s structure decides its function, we know that TKI performs its anticancer function by blocking the ATP-binding site of RTK and then inhibiting the downstream signaling pathway, as well as cell proliferation and differentiation. Coincidently, ABC transporters happened to have two NBDs where could serve as ATP-binding pockets. It is an exciting and challenging assumption whether TKIs would be functioned as chemosensitizers in MDR cancer cell by conjugating to ATP binding site and inhibiting ABC transporter’s function of discharging anticancer drug out of MDR cells (Fig. 3) [73, 74]. A large number of studies focused on the interaction of TKI and ABC transporter is undergoing, including in vitro, in vivo and ex-vivo experiments.

#### ABC transporter modulated by TKIs in vitro and vivo

##### Imatinib (Gleevec, STI571)

Imatinib, targeting BCR-ABL tyrosine kinase, was approved to used for chronic myelogenous leukemia harboring Philadelphia chromosome-positive (Ph+) and gastrointestinal stromal tumors (GIST) with C-kit mutant gene in 2001 by FDA. Özvegy-Laczka et al. reported STI571 exhibits a strong inhibitory effect on ABCG2-dependent dye extrusion at relatively low concentrations, with half-maximal inhibitory effects (IC_50_) were observed at about 0.9 μM, suggesting a high-affinity interaction of ABCG2 with imatinib [75]. A study conducted by Houghton et al. found that overexpression of ABCG2 resulted in a significant increase in resistance (12-fold) to topotecan while imatinib mesylate functioned as a inhibitor to reverse ABCG2-mediated resistance to topotecan by increasing accumulation of topotecan only in cells abundantly expressing ABCG2 (*P* < 0.001) [76]. Sims et al. reported that imatinib could resensitize cancer cells to doxorubicin by inhibiting upregulation of the ABCB1 which results in accumulation of doxorubicin [77].

##### Nilotinib (Tasigna, AMN107)

Nilotinib, a selective BCR-ABL kinase inhibitor, was approved to apply in cases of CML resistant to treatment with imatinib by FDA in 2007. Nilotinib was reported to ameliorate the anticancer response of paclitaxel in the ABCB1- and ABCC10-xenograft, and doxorubicin in a ABCG2-xenograft models [78]. Zhou et al. used MDR models to evaluate the function of nilotinib, showing that at concentrations of 0.75, 1.5 and 3 μM, nilotinib initiates effective reversal of resistance to doxorubicin (27-fold, 81-fold and 141-fold, respectively) in MG63/DOX cell line. What’s more, in nude mouse MDR xenograft models, combination of nilotinib and doxorubicin hampered tumor growth compared with those treated doxorubicin alone (*P* < 0.05), indicating nilotinib potently reverses ABCB1-mediated resistance to doxorubicin both in vitro and vivo [79]. Note that a study conducted by Chen et al. compared BrTet treatment alone and the combination of nilotinib and BrTet in K562/A02 cells, thus found the latter (IC_50_) significantly decrease [80], suggesting the potential function of nilotinib as chemosensitizer.

##### Dasatinib (Sprycel)

Dasatinib, a kind of BCR-ABL kinase inhibitor, was approved to be applied in CML when suffering imatinib treatment failure by FDA in 2006. A study revealed that though dasatinib inhibited ABCG2 less potently than imatinib and nilotinib, but it significantly affected the transportation mediated by ABCB1 at higher micromolar concentrations in Murine HSCs [81]. Hegedűs et al. reported in Sf9 insect cell membranes overexpressing ABCB1 or ABCG2, dasatinib inhibited the efflux of Hoechst 33,342 dye when applied at high concentrations [82], indicating dasatinib’s role as one of ABC transporter inhibitors.

##### Gefitinib (Iressa, ZD1839)

Gefitinib, an EGFR inhibitor, was approved to act as a drug applied in breast, lung and other cancers by FDA in 2003. Leggas et al. found that the accumulation of Hoechst 33,342 dye or calcein dye was higher in the parent cell line than the ABCB1- and ABCG2-overexpessing one with a dose-dependent enhanced by gefitinib [83]. What’s more, gefitinib examined by Özvegy-Laczka et al. exhibited a significant inhibitory effect on ABCG2-dependent Hoechst dye extrusion at low concentration [75], which means gefitinib potently modulated ABC transporter and increased intracellular concentration.

##### Lapatinib (Tykerb, Tyverb)

Lapatinib, a dual tyrosine kinase inhibitor which interrupts the HER2/neu and EGFR pathways, was approved to be prescribed for breast cancer and other solid tumors by FDA in 2007. A study showed that lapatinib at 0.625, 1.25 and 2.5 μM, dose-dependently decreased the IC_50_ of docetaxel, paclitaxel, vinblastine and vinorelbine in HEK-MRP7–2 cells, enhancing these drugs’ accumulation significantly by blocked their efflux [84]. Moreover, it is reported that lapatinib at 2.5 μM could significantly sensitize ABCC1-overexpressing C-A120 cells to its substrate agents such as doxorubicin and vincristine, but not in a non-ABCC1 substrate agent such as cisplatin. Besides, in ABCC1-overexpressing C-A120 cell nude mice xenograft models, significant inhibition of tumor growth was observed in the group with a combination of lapatinib and vincristine compared with control groups (*P* < 0.05) [85]. Similarly, some studies concluded that lapatinib reverses ABCB1- and ABCG2-mediated MDR by directly inhibiting their transport function, contributing to the possibility of co-administration with lapatinib treated MDR cancer patient in clinic [86].

##### Erlotinib (Tarceva, OSI774)

Erlotinib, targeted EGFR, got FDA approval to treat NSCLC in 2004. A study investigating the interaction of erlotinib with selected ABC drug transporters reveals that erlotinib at 2.5 μM slightly decreased the IC_50_ values of colchicine, vinblastine, and paclitaxel in KB-C2 cells and partially reversed their resistance while at 10 μM lowered these values more significantly and reversed most of their resistance [87]. Shi et al. reported that erlotinib increase the intracellular accumulation of [3H]-mitoxantrone in ABCG2-overexpressing cells and became more pronounced with increasing concentrations [88], indicating erlotinib’s potential chance of combinational prescription.

##### Sunitinib (Sutent, SU11248)

Sunitinib, regarded as inhibitor of PDGFR and VEFGR, was approved by FDA for the treatment of renal cell carcinoma and imatinib-resistant GIST in 2006. Dai et al. found that the concentration required to inhibit the growth of S1-M1–80 cells by 50% for topotecan or doxorubicin decreased when combined with sunitinib in contrast with topotecan or doxorubicin alone, which suggests sunitinib potently reverses ABCG2-mediated resistance to topotecan and doxorubicin in vitro [89]. In addition, a research said that the presence of sunitinib slightly reversed ABCB1-mediated resistance to depsipeptide and significantly reversed resistance to topotecan and SN-38 in ABCG2-expressing cells, hinting that sunitinib may be more effective in inhibiting the function of ABCG2 than ABCB1 [16].

#### ABC transporter modulated by TKIs in ex vivo

##### Alectinib (Alecensa)

Alectinib, an inhibitor of ALK, was approved by FDA for the treatment of NSCLC in 2015. In order to investigate whether alectinib could reverse ABCB1-mediated MDR in ex vivo, Yang et al. collected ABCB1-overexpressing bone marrow samples from 4 resistance patients with AML or CML, and found that alectinib potently resensitized these drug resistant samples to Rhodamine 123, doxurubin and verapamil through MTT assays analysis [90], suggesting alectinib is able to reverse ABCB1-mediated MDR phenotype in primary leukemia cell.

##### Ibrutinib (Imbruvica)

Ibrutinib, identified as an inhibitor of BTK, was approved in 2013 to applied in CLL patients by FDA. In an ex-vivo experiment conducted by Zhang et al., they gathered several samples displayed detectable expression of ABCC1 derived from AML or ALL patients and employed them to identify whether ibrutinib could function as an inhibitor of ABC transporter as well as BTK. The result showed that with 5 μM ibrutinib would sensitize these ABCC1-overexpressing samples to vincristine, indicating the co-administration of ibrutinib and vincristine might have potential clinical value [91].

##### Neratinib (Nerlynx, HKI-272)

Neratinib, an dual inhibitor of EGFR and HER2, was approved to prescribe in breast cancers by FDA in 2017. In 2012, Zhao et al. performed flow cytometric analysis to demonstrate the sensitization effect of neratinib in ex-vivo models of ABCB1-overexpressing primary leukemia blasts. Firstly, they obtained clinical samples of ABCB1-overexpressing leukemia cells from patients. Then they tested the influence of neratinib on intracellular Rhodamine 123 accumulation. Finally, they found that neratinib would increase the intracellular Rhodamine concentration with a dose-dependent manner (0.25–1.0 μM). What’s more, the MTT cytotoxicity assays showed that neratinib markedly sensitized primary leukemia blasts to doxurubin compared with the control group (*P* < 0.05), indicating neratinib may play a role in the reversal of ABCB1-mediated MDR phenotype [92].

##### Osimertinib (Tagrisso, Tagrix)

Osimertinib, a third generation of EGFR TKI drug approved by FDA in 2015, was applied in metastatic NSCLC patient. So as to explore whether osimertinib could reverse ABCB1-mediated MDR in ex vivo, Chen et al. Collected bone marrow samples which highly expressed ABCB1 from patients diagnosed with AML and performed flow cytometric analysis to examine the effect of osimertinib on intracellular accumulation of Rhodamine 123 afterwards. In accordance with expectation, osimertinib could increase the intracellular Rhodamine 123 concentration. In addition, MTT assays analysis showed that osimertinib significantly exhibited its reversal efficiency at 0.4 μM concentration [93].

Besides those mentioned above, dozens of TKIs also were documented to act as inhibitors of ABC transporters in vivo, in vitro and ex vivo, including but not limited to axitinib, trametinib, saracatinib, EKI785, quizartinib, bosutinib, afatinib, apatinib, ponatinib, nintedanib, AG1478, AST1306, canertinib, cediranib, icotinib, ceritinib, telatinib, sorafenib, motesanib, masitinib, linsitinib, PD173074, vemurafenib, vandetanib,WHI-P154, crizotinib, GW583340, GW2974, regorafenib, CEP-33779, cabozantinib, tandutinib, vatalanib. Due to the limited scope, we show them in the form of table as follow (Table 3).

**Table 3:** TKIs function as inhibitors of ABC transporters

TKIs	Target	ABC transporter inhibitor	Applications	Approved by FDA	Reference
Afatinib	EGFR,HER2	ABCG2	NSCLC	2013	[107]
AG1478	EGFR	ABCB1, ABCG2	NA	Not approved	[108]
Alectinib	ALK	ABCB1, ABCG2	NSCLC	2015	[90]
Apatinib	VEGFR	ABCB1, ABCG2	Gastric carcinoma	Not approved	[109]
AST1306	EGFR	ABCG2	NA	Not approved	[110]
Axitinib	VEGFR, PDGFR	ABCG2	RCC	2012	[111]
Bosutinib	BCR-ABL,Src	ABCG2	CML	2012	[82]
Cabozantinib	VEGFR, Kit	ABCG2	Thyroid cancer	2011	[112]
Canertinib	EGFR	ABCB1, ABCG2	NA	Not approved	[113]
Cediranib	VEGFR	ABCB1, ABCC1	NA	Not approved	[114]
CEP-33779	JAK	ABCB1	NA	Not approved	[115]
Ceritinib	ALK	ABCB1, ABCG2	NSCLC	2014	[116]
Crizotinib	ALK	ABCB1	NSCLC	2011	[117]
Dasatinib	BCR-ABL, Src	ABCB1, ABCG2	CML	2006	[81]
EKI785	EGFR	ABCB1, ABCC1	NA	Not approved	[94]
Erlotinib	EGFR	ABCB1, ABCG2, ABCC10	NSCLC	2004	[84, 87]
Gefitinib	EGFR	ABCB1, ABCG2	NSCLC	2003	[75, 118]
GW2974	EGFR	ABCB1, ABCG2	NA	Not approved	[119]
GW583340	EGFR	ABCB1, ABCG2	NA	Not approved	[119]
Ibrutinib	BTK	ABCC1	Lymphoma	2013	[91]
Icotinib	EGFR	ABCG2	NSCLC	2011	[120]
Imatinib	BCR-ABL	ABCB1, ABCC1, ABCG2, ABCC10	CML, GIST	2001	[76, 77, 88, 94]
Lapatinib	HER2, EGFR	ABCB1, ABCC1, ABCG2, ABCC10	Breast cancer	2007	[84–86]
Linsitinib	IGF	ABCG2, ABCC10	NA	Not approved	[121]
Masitinib	Kit	ABCG2, ABCC10	Mast cell tumor	Not approved	[122, 123]
Motesanib	VEGFR	ABCB1, ABCG2	NA	Not approved	[124]
Neratinib	HER2, EGFR	ABCB1	Breast cancer	2017	[92]
Nilotinib	BCR-ABL	ABCB1, ABCG2, ABCC10	CML	2007	[78]
Nintedanib	VEGFR, PDGFR	ABCB1	NSCLC	2014	[125]
Osimertinib	EGFR	ABCB1, ABCG2	NSCLC	2015	[93]
PD173074	VEGFR	ABCB1, ABCC10	NA	Not approved	[126, 127]
Ponatinib	BCR-ABL	ABCB1, ABCG2, ABCC10	CML	2012	[128, 129]
Quizartinib	FLT3	ABCG2	AML	Not approved	[130]
Regorafenib	VEGFR	ABCB1	GIST	2012	[131]
Saracatinib	Src	ABCB1	NA	Not approved	[132]
Sorafenib	VEGFR, PDGFR	ABCB1, ABCC2, ABCC4, ABCG2	RCC, HCC	2005	[133]
Sunitinib	VEGFR, PDGFR	ABCB1, ABCG2	GIST, RCC	2006	[16, 89]
Tandutinib	FLT3	ABCG2	NA	Not approved	[134]
Telatinib	VEGFR	ABCG2	NA	Not approved	[135]
Trametinib	MEK	ABCB1	Melanoma	2013	[136]
Vandetanib	VEGFR, EGFR	ABCB1, ABCC1, ABCG2	Thyroid cancer	2011	[137, 138]
Vatalanib	VEGFR	ABCB1, ABCG2	Colorectal cancer	Not approved	[139]
WHI-P154	JAK	ABCG2	NA	Not approved	[140]

#### TKIs’ potential mechanism to reverse MDR

**Fig. 4:**
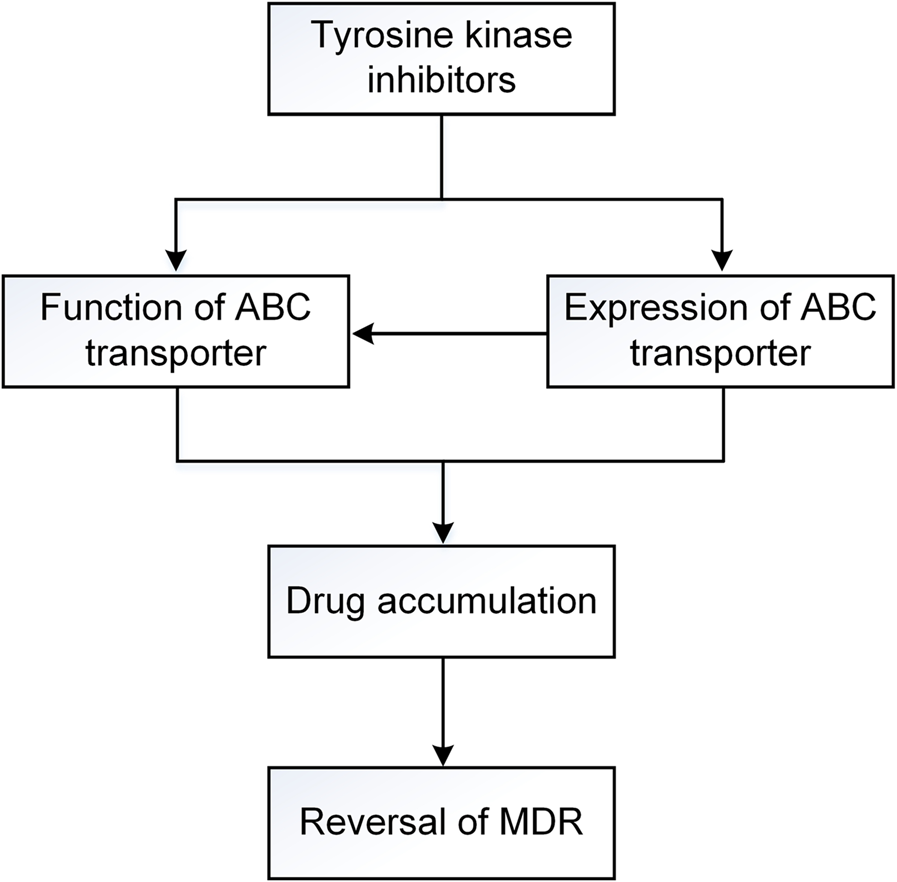
TKIs’ potential mechanism to reverse MDR. TKIs inhibit ABC transporter in manner of: (i) directly inhibiting the function of ABC transporter through blocking the ATP-binding site or changing the single nucleotide polymorphisms (SNPs) (ii) down-regulating the expression of ABC transporter and consequently influencing its normal function. Which increases intracellular drug concentration and results in reversal of MDR

According to the experiments and analysis we mentioned above, the role of TKIs functioned as ABC transporters inhibitors is undoubtedly clarified. On the other hand, it also provides an evidence that different TKIs inhibit their own ABC transporter and moreover, not all of TKIs has reversal efficiency by modulating ABC transporter. Here, we have to admit that the specific mechanism how TKIs reverse MDR phenotype is still unclear due to lack of integral related literature and comprehensive research describing Structure Activity Relationships (SAR) between TKIs and ABC transporters. From current literature, we conclude the potential ways as follows (Fig. 4): (i) blocking the ATP-binding site of ABC transporter. It had been identified that TKI could exhibit its function by blocking the ATP-binding site of RTK and then interfering the downstream signaling transduction. Coincidently, ABC transporters happened to have two NBDs where could serve as ATP-binding pockets. To prove whether TKI would inhibit ATP-binding site of ABC transporter, a study conducted by Hegedűs et al. showed that the activation of the MDR1-ATPase stimulated by verapamil is significantly inhibited by STI571 and EKI785 in different concentration [94], which backs our assumption. (ii) down-regulating the expression of ABC transporter. It is said that TKIs potently influence the re-localization and expression of ABC transporters by inhibiting the PI3K-Akt or Raf-MEK-ERK pathway [95, 96]. For instance, In SGC7901/DDP cell lines, expression levels of MDR1, p-Akt, and p-ERK were significantly decreased after sorafenib treatment [97]. (iii) changing the single nucleotide polymorphisms (SNPs) in ABC transporters. Au et al. was aimed at the relationship between SNPs of ABCB1 and imatinib-resistance in chronic myelocytic leukemia patients, suggesting the TKIs is likely to change the SNPs of ABC transporter to develop drug resistance [98]. It is conceived that TKIs alter SNPs of ABC transporter to reverse drug resistance, which overlaps similar opinion to another review [71].

### Clinical trials about TKIs enhanced conventional chemotherapeutics

In contrast to a large number of in vitro, in vivo and ex-vivo experiments aforementioned, quite a few clinical trials focused on whether TKIs enhance the efficacy of conventional chemotherapy are documented due to the its complexity and side-effect when applied in human, along with only several of them received positive outcomes (Table 4).

**Table 4:** Clinical trials of co-administration regimen in resistant patients

Combinational strategy	Malignancy	Reference/Identifier
Erlotinib+Gemcitabine	Pancreatic cancer	[100, 101]
Lapatinib+Capecitabine	Breast cancer	[102–104]
Nintedanib+Docetaxel	NSCLC	[106]
Erlotinib+Everolimus	Head and Neck squamous cell carcinoma	[141]
Erlotinib+Carboplatin	Ovarian cancer	[142]
Erlotinib+Topotecan	Solid tumor	[143]
Lapatinib+Epirubicin	Breast cancer	NCT 00753207
Sorafenib+Paclitaxel	Solid tumor	NCT 00572078

Gemcitabine alone became the first-line treatment for pancreatic cancer decades ago. Until now, an increasing number of evidences showed that cancer cells had developed drug resistance to it [99]. To overcome the resistance, a study conducted by Moore et al. revealed that in pancreatic cancer, overall survival was significantly longer in the erlotinib/gemcitabine arm compared with gemcitabine alone arm with an estimated HR of 0.82 (95%CI: 0.69–0.99, *P* = 0.038). Median survival times were 6.24 months versus 5.91 months for the erlotinib/gemcitabine versus placebo/gemcitabine groups with 1-year survival rates of 23% (95%CI: 18%–28%) and 17% (95%CI: 12%–21%), respectively (*P* = 0.023), suggesting erlotinib significantly enhanced the efficacy of gemcitabine in pancreatic cancer [100]. A systematic review with meta-analysis accomplished by Yang et al. concluded that gemcitabine plus erlotinib represent a new option for the treatment of advanced pancreatic cancer, with modest but clinically meaningful compared gemcitabine alone [101].

In 2007, FDA approved the co-administration of lapatinib and capecitabine in HER-2-overexpressing metastatic breast cancer who had received but failed the prior therapy including anthracycline, taxane and trastuzumab [102]. In order to evaluating the efficacy of this combination, a phase III study conducted by Geyer et al. showed that lapatinib plus capecitabine improved time to progression (8.4 months) compared with capecitabine alone (4.4 months, *P* < 0.001) [103]. What’s more, a study initiated by Cetin et al. recruited 203 patients who were in the condition of HER2-positive metastatic breast cancer progressing after trastuzumab and chemotherapy including anthracycline and taxane, and treated them with the combination of lapatinib and capecitabine. Among all 203 patients, there were 7 complete responses (CRs), 61 partial responses (PRs) and 77 stable diseases (SDs). The median PFS was 7 months (95%CI: 6–10 months) while median OS was 15 months (95%CI: 12–18 months), indicating lapatinib and capecitabine combination therapy is effective in these patients [104].

Docetaxel, as we know, is approved to be administrated as a treatment for numerous cancers. Unfortunately it is abundantly reported to develop drug resistance in recent years [105]. To handle this resistance, a study conducted by Reck et al. found that in this population of patients with adenocarcinoma who had progressed after first-line therapy, median PFS was significantly longer in the docetaxel plus nintedanib group compared docetxel alone, both at the time of the primary PFS analysis (*P* = 0.0008) and final overall survival analysis (*P* = 0.0005) [106], identifying the dramatic efficacy of the combination of nintedanib and docetaxel in patients with advanced NSCLC progressing after the failure of first-line chemotherapy.

Despite the several successful combination mentioned above, most trials did not focus on the reversal of ABC transporter-mediated MDR. In the last decade, a huge amount of effort has been invested in the field of ABC drug transporters to identify, develop, and clinically evaluate a variety of agents known to antagonize the function of these transporters as a means of overcoming tumor resistance. The major reasons for the failure of this strategy could be explained in retrospect by multiple factors and variable components that are involved in the development of drug resistance in patients. We advocate further study on combination of TKI (such as afatinib, belongs to 3rd generation MDR modulator) and conventional chemotherapy in clinic in the patients with ABC-transporter expression. Patient selection for clinical studies is a key factor. Patients whose tumors express high levels of ABC-transporter will obviously receive the most benefit from modulators. Therefore, drug-resistance reversal trials should ideally be performed in individuals with tumors that initially are chemosensitive but develop drug resistance following initial therapy, which is marked by an increase in the expression of ABC drug transporter.

## Conclusion and perspective

With multidrug resistance prevails, the prescription of chemotherapeutics alone becomes increasingly useless and impracticable. We have to realize that there is a irresistible trend to develop combinational strategies regarding MDR phenotype. The recent study have provided evidences that TKIs can reverse MDR by blocking the function of ABC transporter and subsequently promote drug accumulation. Co-administration of TKIs with other conventional chemotherapeutics is proven to be a feasible alternative in MDR cancer cells which is supported by in vivo, in vitro, ex-vivo experiments and clinical trials. However, combination strategies in clinic would not always receive satisfying outcomes partly because of the unclear reversal mechanism and a lack of suitable patients. Further studies are still indispensable to clarify its mechanism and unveil more effective combinational strategies in clinic.

## Abbreviation

ABC transporter: ATP-binding cassette transporter
ABCB1: ATP binding cassette sub-family B member 1
ABCC1: ATP binding cassette sub-family C member 1
ABCG2: ATP binding cassette sub-family G member 2
ALK: ATP-competitive anaplastic lymphoma kinase
AML: Acute myelogenous leukemia
BCR-Abl: Breakpoint cluster region-abelson complex
B-Raf: V-Raf murine sarcoma viral oncogene homolog B
BTK: Bruton’s tyrosine kinase
CLL: Chronic lymphocytic leukemia
CML: Chronic myelogenous leukemia
EGFR: Epidermal growth factor receptor
ERK: Extracellular signal-regulated kinases
FLT3: FMS-like tyrosine kinase
GBM: Glioblastoma multiforme
GIST: Gastrointestinal stromal tumors
HCC: Hepatocellular carcinoma
HER2: Human epidermal growth factor receptor 2
IGF: Insulin-like growth factor
JAK: Janus kinase
Kit: Mast/stem cell growth factor receptor kit
MAPK: Mitogen-activated protein kinase
MEK: Mitogen-activated protein kinase
NRTK: Non receptor tyrosine kinase
NSCLC: non-small cell lung cancer
PDGFRs: Platelet-derived growth factor receptors
PDGFs: Platelet-derived growth factors
PI3K: Phosphatidylinositol-3-kinase
Raf: Rapidly accelerated fibrosarcoma
RCC: Renal cell carcinoma
RTK: Tyrosine kinase receptor
Src: Proto-oncogene tyrosine-protein kinase Src
TKIs: Tyrosine kinase inhibitors
TKs: tyrosine kinases
VEGFR: Vascular endothelial growth factor receptor
